# Novel Electrothermal Microgrippers Based on a Rotary Actuator System

**DOI:** 10.3390/mi13122189

**Published:** 2022-12-10

**Authors:** Pedro Vargas-Chable, Margarita Tecpoyotl-Torres, Gerardo Vera-Dimas, Volodymyr Grimalsky, José Mireles García

**Affiliations:** 1 Instituto de Investigación en Ciencias Básicas y Aplicadas-Centro de Investigación en Ingeniería y Ciencias Aplicadas (IICBA-CIICAp), Universidad Autónoma del Estado de Morelos, Cuernavaca 62209, Mexico; 2 Facultad de Ciencias Químicas e Ingeniería (FCQeI), Universidad Autónoma del Estado de Morelos, Cuernavaca 62209, Mexico; 3 Centro de Investigación en Ciencia y Tecnología Aplicada, Universidad Autónoma de Ciudad Juárez, Av. Plutarco Elías Calles #1210, Fovissste Chamizal, Ciudad Juárez 32310, Mexico

**Keywords:** microgripper, V-shaped, rotary actuator, Ansys^TM^, FEM, MEMS, electrothermal actuation

## Abstract

Microgrippers are devices that have found applications in various fields of research and industry. They are driven by various actuation methods. In this article, an electrothermal rotary actuator recently proposed in the literature is explored to obtain a novel microgripper design (Model 1). In addition, the use of the rotary actuator as part of the chevron actuated microgrippers (Model 2) is also discussed. The theoretical analysis of the rotary actuator is supported by an equivalent U-shaped-like microactuator. The small error values validate the approximation used. Numerical modeling is performed with ANSYS^TM^ (Student version 2022, ANSYS, PA, USA). A comparison of theoretical and numerical results provides acceptable error values. The total inter-jaw displacement values obtained for models 1 and 2 are 12.28 μm and 21.2 μm, respectively, and the reaction force is 8.96 μN and 34.2 μN, respectively. The performance parameters of both microgrippers could make their use feasible for different nanoapplications. Model 2 can be used when higher force and displacement are required.

## 1. Introduction

Microelectromechanical systems (MEMS) are classified as sensors and actuators. They have awakened a wide interest in various areas of research, both at institutional and business levels, due to their wide applications in daily life and their impact on social welfare. Their use has improved the level of comfort in various areas, such as automotive and residential, and has allowed for the monitoring of environmental variables, improving safety conditions, among many other applications, which are constantly emerging. Recently, new, or optimized microactuators [1,2] have been reported, such as micropositioners [3,4], microswitches [5], microgrippers [6,7], piezoelectric devices [8,9], microgenerators [10,11], micropumps [12], and bimorph actuator [13,14]. 

Microgrippers are widely used in different research fields, including health, biology, chemistry, materials science, etc., as well as in manufacturing processes, where microassembly is a fundamental task. According to the characteristics of the clamping object, several geometries and microfabrication technologies have been proposed in the literature, as shown in Table 1. 

A review of different microgrippers for manipulation and assembly of microwires to Printed Circuit Board (PCB) connectors is provided in [15], where the two major classifications based on their structures are also given: cantilever and flexible hinge. In addition, the main advantages of each microgripper’s group are presented. For the case of microgrippers with cantilever type structures, among their main advantages are their ease of manufacture and the large displacement between their tips with larger dimensions [16,17]. The main disadvantages provided are the difficulty in scaling them down to lower microscales, their jaw tips not consistently being parallel, and their increased arm length, resulting in an increase in jaw aperture size but a decrease in applied force. The actuation types reported for these microgrippers are electromagnetic, piezoelectric, electrostatic, electrothermal, and shape memory alloy.

On the other hand, in [17] a micro-tweezer based on a pneumatically actuated cantilever is reported. The actuator is essentially a flexible membrane that applies force to the gripper pad when the air inlet is pressurized, producing arm or cantilever deflection. The output force is in the order of 450 mN, whereas the input force is 48 mN. The tests were performed manipulating acid-washed zirconium microbeads of 200 μm diameter in air and underwater.

In this work, the interest focuses on the microgrippers based on the cantilever type structure because the architecture of the proposed prototype has cantilevers as part of its jaws and as essential parts of its actuator arrangement. The research is focused on demonstrating that a microgripper can be developed from a rotational microactuator, taking advantage of the thermal effects on the structure, mainly from the deformation and buckling effect of beams, allowing for the development of a complex device from simple structural elements.

As can be seen in Table 1, no microgripper was found to have any similar rotary microactuator, so part of the novelty is its implementation and use. It is noteworthy that this rotary actuator arrangement was previously reported [18], where the dimensions of the microactuator and the microgripper Model 2 were optimized by simulation. In addition, the theoretical model of the actuator of microgripper Model 1 is proposed. It should be noted that the individual rotary actuator has been reported in [1].

**Table 1:** State-of-the-art of recent microgrippers, their actuators, and fundamental parameters.

Ref.	Microgripper Type	Microactuator Type	Structural Material	Software for Simulation	Dimensions (µm)	Feed	Displacement of Tips (µm)	Initial Gap (µm)	Force on Tips (µN)	Stress (MPa)
[7]	Electrothermal	U-Beam	Silicon	Gmsh and MATLAB	NA	397.5 °K	≈2.6	NA	NA	NA
[19]	Electrothermal	Hot arms	SU-8 and Cr/Au/Cr-based	ANSYS^TM^ Solid 98	≈1000 × 140 × 20	180 °C at 650 mV	≈50.5 and 47.8	30.5 and 30.1	NA	NA
[20]	Compliant	External actuation	SU-8	ANSYS^TM^	≈1000 × 390 × 20	NA	≈35	10	≈25	32.94
[21]	Electrostatic	Comb drive	NA	FEA	NA	80 V	≈30	NA	≈140 to 160	NA
[22]	Electrothermal	U-Beam	Polysilicon	CoventorWare^TM^	≈400 × 42 × 2	5 V	15	5	NA	NA
[23]	Electrothermal	Beams	SU-8 and Au	NA	≈1300 × 610 × 10	0.65 V and 0.7 V	11 and 8	40	NA	NA

Note: Not Available (NA). Finite Element Analysis FEA

In Section 2, the design, modeling, and simulation of the proposed microgripper models are shown. In Section 3, results obtained from analytical and numeric models are given and compared, for both microgripper models, as well as with some other microgrippers. The corresponding discussion is also given in Section 3. Section 4 provides some details about the feasibility of the microgrippers’ fabrication. Finally, some concluding remarks are provided in Section 5.

## 2. Materials and Methods

### 2.1. Design Concept and Simulation 

**Figure 1:**
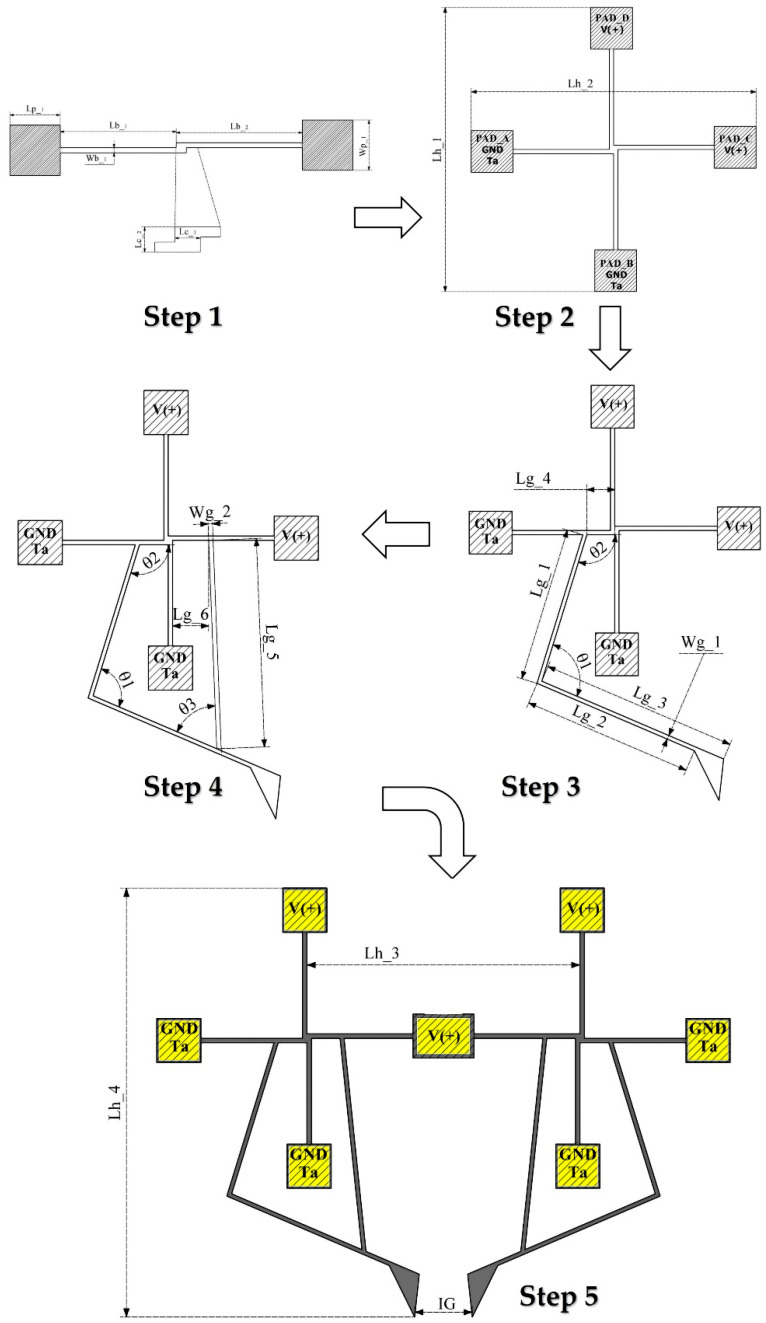
Design flow of the normally open electrothermal microgripper Model 1, considering the evolution steps of the schematic diagram.

Microcantilevers [24,25] are widely used and studied devices. These microelements can be thermally, electrically, mechanically, optically, or magnetically powered. They are fundamental parts of simple and complex systems. In this work, a micro electrothermal actuator designed with silicon and based on an array of four orthogonally distributed cantilever beams is used. The guided ends of the beams are joined at the central junction point, while the fixed ends are connected to a positive or negative potential, according to Figure 1, where in the first block the construction sequence of the rotary microactuator is observed [18]. A similar structure is given in [1] using polysilicon and silicon, respectively. This actuator is called rotary according to the shape of the displacement generated in the beams under polarization. Table 2 describes the geometrical variables showing the dimensions of the microactuator design and of the microgripper, in accordance with Figure 1. 

In the case of two beams (first step, Figure 1), the polarization is performed with a potential difference, positive in one of the pads and 0 V in the other one, assigning the room temperature in the pad considered as the electrical ground. For the 4-arm microactuator, 2 arms are fed with a positive potential and the other two are grounded, the last assigned the ambient temperature, as shown in the second step of Figure 1. In step 3, one of the two sections of the gripper, consisting of an arm and a 4-beam actuator, is shown. In step 4, a damping arm element is added to the corresponding arm.

The design of the complete normally open microgripper (Figure 1, step 5) is made by joining both symmetrical sections of the microgripper. The two actuators form a new arrangement, which shares a central anchor, forming a new 7-beam microactuator, 3 of which are fed with the positive potential and 4 of which are grounded; in the last ones, the ambient temperature is also assigned.

The positive potential-fed beams deform thermally in a directional manner, allowing buckling, generated by the Joule effect, favored by the constraint generated by the central joint of each array. The electrically grounded trampolines also exhibit electrothermic deformation. The buckling occurs in opposite directions in each array, causing the gripper arms, additionally supported by the damping elements, to move in a symmetrical and stable manner, causing the jaws to close. When the positive potential is removed, the gripper arms return to their original positions.

**Figure 2:**
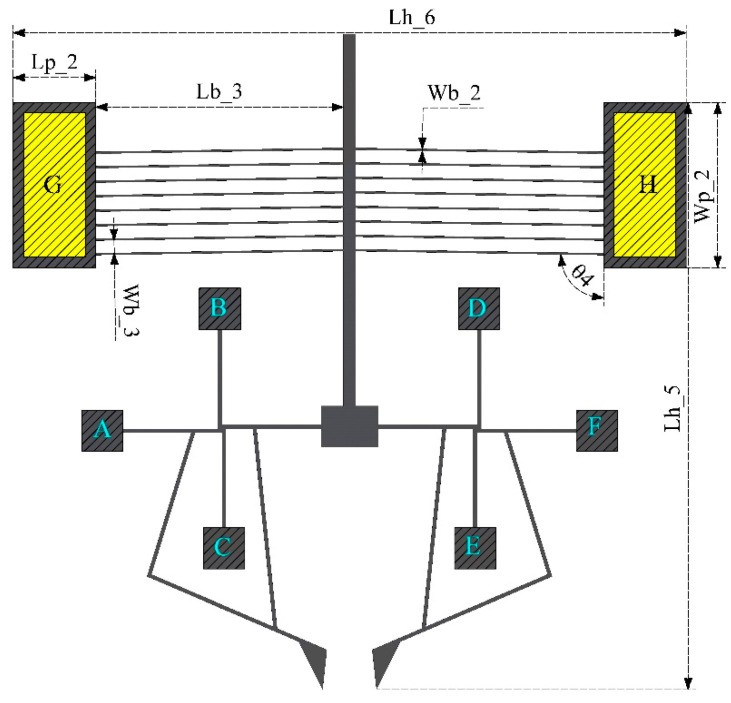
Microgripper Model 2, electrochemically actuated by chevron.

The microgripper proposed can be alternatively driven by a chevron actuator (case Model 2), as shown in Figure 2, where the central connection anchor of the two rotary actuators is now considered as a sliding joint mass driven by the chevron. The remaining anchors (A, B, C, D, E, and F) will be considered in this case as mechanical anchors. In this case, the deformation of the orthogonal arrays is like Model 1. 

Table 3 shows the electrical, mechanical, and thermal parameters of silicon (Si) considered in the simulations with ANSYS^TM^ (Student version 2022, ANSYS, PA, USA) as well as in the development and evaluation of analytical models. The geometric dimensions of the microgripper elements are taken from Table 2. The electrothermal actuators for microgripper designs 1 and 2 will be fed with 2 V. The thickness of the structure was determined according to the SOI wafers used in the process in which they could be fabricated.

In the following section, the equations that theoretically validate the relevant parameters of the orthogonal and chevron microactuators, for design cases 1 and 2, respectively, are developed. The force and displacement generated by the orthogonal actuators with 2 and 4 beams were analyzed, for case 1, where the theory of the U-beam microactuators was adapted, i.e., considering hot and cold beams. For design 2, the equations corresponding to the parameters that characterize the performance of the chevron actuator are shown, mainly those of force, displacement, stress, electric current intensity, and power.

### 2.2. Modelling of Microactuators 

#### 2.2.1. Thermal Elongation of Beams

In this subsection, the equations are provided that model the electrothermal behavior of the microactuator consisting of two beams joined at their guided ends, by means of a small step, while their opposite ends are fixed by means of an anchor, respectively, as can be seen in step 1 of Figure 1. 

The thermally induced elongation of a differential segment of each beam is given by [26]:

(1) $$dL=\alpha \left(T-Ta\right)dS$$

The total displacement of each beam is obtained by integrating the differential elongation of the beam as follows:

(2) $$\Delta L=\int_{0}^{L}dL=\int_{0}^{L}\alpha \left(T-Ta\right)dS=\alpha \Delta TL$$

The force is obtained combining the expressions of stiffness and displacement:

(3) $$F=\frac{EA}{L}\Delta L\equiv k_{e}\Delta L$$

where *F* is the actuating force of the device due to elongation generated by the thermal effect, ∆*L* is the deformation of the beams due to the Joule effect, and ∆*T* is the temperature difference between the ambient temperature and the temperature generated by applying the voltage source to the actuator’s pads.

#### 2.2.2. Electromechanical Modeling of Two Beams to a U-Beam-like Microactuator

**Figure 3:**
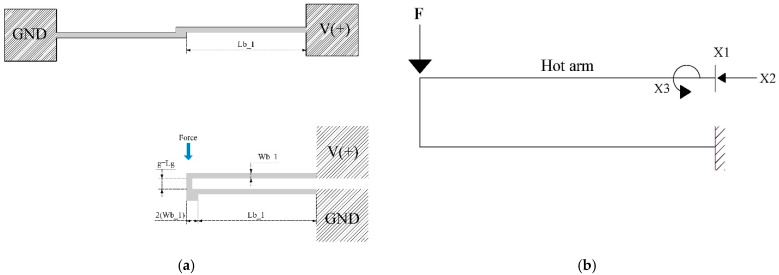
(**a**) Microactuator of two beams (MTB) transformed as a U-shaped beam microactuator, and (**b**) simplified rigid frame of the thermal actuator with three redundant elements.

For the electromechanical modeling, the validation of the microactuator with two horizontal beams was carried out taking as a reference the model of a U-shaped beam microactuator, as can be seen in the diagrams shown in Figure 3.

The transformation and equivalence, as well as the modeling of a two-beam microactuator to a U-beam-like microactuator, are based on [1,27], respectively. In Equation (4), the initial behavior of the rotary microactuator is defined. It is important to mention that the force applied at the free end of the device is the force obtained by applying an electric potential difference [7].

Applying the matrix force method for *X*_1_, *X*_2_, and *X*_3_, the general equation describing the displacement of the microactuator can be obtained with Equation (4):

(4) $$\left[\begin{array}{ccc} f_{11} & f_{12} & f_{13}\\ f_{21} & f_{22} & f_{23}\\ f_{31} & f_{32} & f_{33} \end{array}\right]\left[\begin{array}{c} X_{1}\\ X_{2}\\ X_{3} \end{array}\right]=\left[\begin{array}{ccc} 0 & 0 & 0\\ \Delta L_{h} & -\Delta L_{c} & -\Delta L_{f}\\ 0 & 0 & 0 \end{array}\right]\;\mathrm{where}\;\Delta L_{c1}=\Delta L_{f},\;\Delta L_{b1}=\Delta L_{c},\;\mathrm{and}\;\Delta L_{b2}=\Delta L_{h}$$

Then:

$$[\begin{array}{c} \frac{1}{3EI_{h}}\left(L_{b2}^{3}+{\left(L_{b1}+W_{b1}\right)}^{3}\right)+\frac{1}{3EI_{c}}\left(3L_{b2}^{2}L_{g}+L_{b2}^{3}-{\left(L_{b1}+W_{b1}\right)}^{3}\right)\\ -\frac{1}{2EI_{h}}\left({\left(L_{b1}+W_{b1}\right)}^{2}L_{g}\right)-\frac{1}{2EI_{c}}\left(L_{g}^{2}L_{b2}+L_{b2}^{2}L_{g}-{\left(L_{b1}+W_{b1}\right)}^{2}L_{g}\right)\\ -\frac{1}{2EI_{h}}\left(L_{b2}^{2}+{\left(L_{b1}+W_{b1}\right)}^{2}\right)-\frac{1}{2EI_{c}}\left(L_{b2}^{2}+2L_{g}-{\left(L_{b1}+W_{b1}\right)}^{2}\right) \end{array}$$

$$\begin{array}{c} -\frac{1}{2EI_{h}}\left({\left(L_{b1}+W_{b1}\right)}^{2}L_{g}\right)-\frac{1}{2EI_{c}}\left(L_{g}^{2}L_{b2}+L_{b2}^{2}L_{g}-{\left(L_{b1}+W_{b1}\right)}^{2}L_{g}\right)\\ \frac{1}{3EI_{c}}\left(L_{g}^{3}+3L_{c1}L_{g}^{2}\right)+\frac{1}{EI_{h}}\left(\left(L_{b1}+W_{b1}\right)L_{g}^{2}\right)\\ \frac{1}{2EI_{c}}\left(L_{g}^{2}+2L_{g}L_{c1}\right)+\frac{1}{EI_{h}}\left(L_{g}\left(L_{b1}+W_{b1}\right)\right) \end{array}$$

$$\left.\begin{array}{c} -\frac{1}{2EI_{h}}\left(L_{b2}^{2}+{\left(L_{b1}+W_{b1}\right)}^{2}\right)-\frac{1}{2EI_{c}}\left(L_{b2}^{2}+2L_{g}-{\left(L_{b1}+W_{b1}\right)}^{2}\right)\\ \frac{1}{2EI_{c}}\left(L_{g}^{2}+2L_{g}L_{c1}\right)+\frac{1}{EI_{h}}\left(L_{g}\left(L_{b1}+W_{b1}\right)\right)\\ \frac{1}{EI_{h}}\left(L_{b2}+\left(L_{b1}+W_{b1}\right)\right)+\frac{1}{EI_{c}}\left(L_{c1}+L_{g}\right) \end{array}\right]\times \left[\begin{array}{c} X_{1}\\ X_{2}\\ X_{3} \end{array}\right]$$

(5) $$=\left[\begin{array}{ccc} 0 & 0 & 0\\ \frac{1}{6E}\left(\frac{L_{c1}^{2}}{I_{c}}\left(2(L_{b1}+W_{b1})+L_{b2}\right)+\frac{{\left(L_{b1}+W_{b1}\right)}^{2}}{I_{f}}\left(2L_{c1}+\left(L_{b1}+W_{b1}\right)\right)\right) & \frac{L_{g}}{2E}\left(\frac{L_{c1}^{2}}{I_{c}}+\frac{L_{b1}+W_{b1}}{I_{f}}\left(L_{c1}+L_{b2}\right)\right) & \frac{1}{2E}\left[\frac{L_{c1}^{2}}{I_{c}}+\frac{L_{b1}+W_{b1}}{I_{f}}\left(L_{c1}+L_{b2}\right)\right]\\ 0 & 0 & 0 \end{array}\right]$$

Therefore, to calculate the redundant elements *X*_1_, *X*_2_, and *X*_3_, as shown in Figure 3b, the matrix equation is solved. The following relationships are applied:

(6) $$X_{1}=F\frac{\Delta_{1}}{\Delta };\;X_{2}=F\frac{\Delta_{2}}{\Delta }\;and\;X_{3}=F\frac{\Delta_{3}}{\Delta }.$$

The deflection of the free section of the microactuator is calculated by the virtual work method, obtaining:

(7) $$u=\int \frac{\bar{M}M}{EI_{h}}ds=\frac{L_{b2}^{2}}{6EI_{h}}\left(X_{1}L_{b2}-3X_{3}\right)$$

The equation that allows one to approximate the displacement results from an external force applied to the end of the U-beam-like microactuator and that analogously models the 4-beam microactuator is:

$$\begin{array}{cc} u=\int \frac{\bar{M}M}{EI_{c}}ds= & \frac{\left(L_{b1}+W_{b1}\right)}{6EI_{f}}\{(\left(2\times L_{c1}\right)+L_{b2})\left[X_{3}+X_{2}L_{g}-X_{1}\left(L_{b2}-L_{c1}\right)\right]+(2L_{b2}\\ & +L_{c1})\left(X_{3}+X_{2}L_{g}\right))\}+ \end{array}$$

(8) $$\frac{L_{c1}^{2}}{6EI_{c}}\left[3\left(X_{3}+X_{2}L_{g}-X_{1}L_{c1}\right)+2X_{1}L_{c}\right]$$

To calculate the stiffness constant of the element, the following equation is used:

(9) $$k_{c}=\frac{3E}{L_{b_{1}}^{3}}\left(\frac{t\times w_{b_{1}}^{3}}{12}\right)=\frac{3E}{L_{b_{1}}^{3}}\times I$$

#### 2.2.3. Electromechanical Modeling of V-Shaped Beam Microactuator

The microgripper can also be electrothermally actuated by means of a V-shaped actuator (Model 2, Figure 2). The coupling is made by connecting the shaft of this device at the junction point of the rotary actuators, whose anchors in this case function only as mechanical anchors. To support the functional description of this model, the equations of displacement and actuating force of the V-shaped actuator are described. 

The V-shaped micro-actuator is a device that is integrated by two fixed elements or anchors, and the fixed extremes of the symmetrical beams are connected to the anchors with an inclination angle. The other beam extremes are joined to the shaft. Thermal expansion of beams generates the shaft displacement. The equation describing the displacement behavior of the device due to temperature increase is given by [28,29]:

(10) $$U_{y}=\frac{\Delta T\alpha L\sin\theta \;}{s^{2}+c^{2}\left(\frac{12I}{A_{c}L^{2}}\right)}$$

where ∆*T* is the temperature difference between ambient temperature and the thermoelectrically generated temperature. $A_{c}$ is the cross-section area, *c =* cos*θ* and *s =* sin*θ*, *L* is the beam length, and *I* is the second inertia moment of the cross-section area of the beams. The thermic energy generated by electric charges generates a unidirectional thermal force, which can be calculated by:

(11) $$F_{b}=A_{c}E\frac{\Delta L}{L_{0}}=A_{c}E\Delta T\alpha$$

where ∆*L* is the increment of the beam length, defined by $\Delta L=L_{0}\Delta T\alpha$, where α is the thermal expansion coefficient, and *E* is Young’s modulus. The beam force due to the thermal effect, and considering the inclination angle, can be obtained from:

(12) $$F_{by}=A_{c}E\Delta T\alpha \sin\theta$$

The stiffness constant *k* can be obtained from Hooke’s Law, *k = F_by_/U_y_.*

### 2.3. Pseudo-Rigid Body Model of Microgripper Model 2

**Figure 4:**
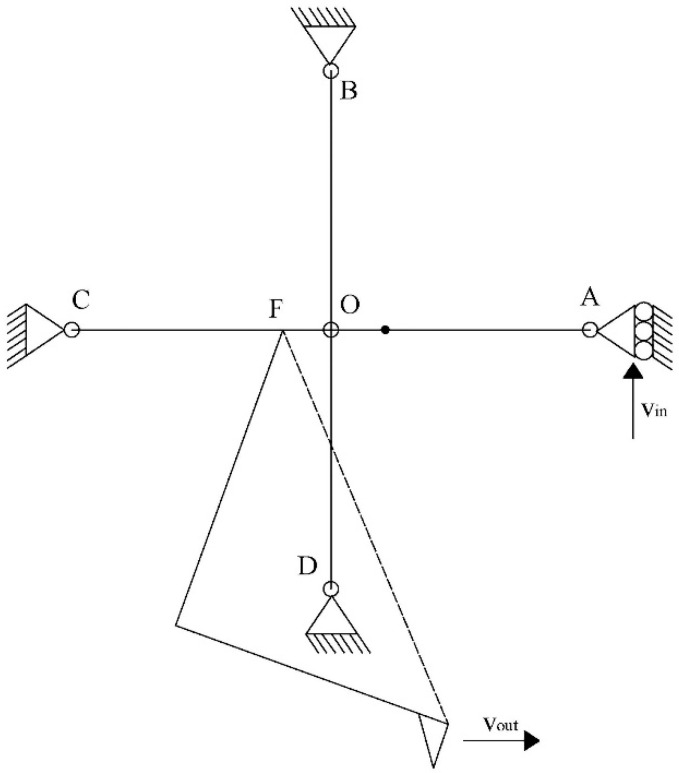
PRBM of a middle microgripper, Model 2.

The displacement modelling of the microgripper Model 2 is obtained by the pseudo rigid body model (PRBM) [30] method. Due to the microgripper symmetry, the model is performed considering a half of it, which includes one arm and one of the rotary actuators. The linkages are considered as rigid bodies and the beam ends as torsional springs. Figure 4 shows the equivalent simplified model of the middle microgripper. The input displacement is provided by the shaft of the chevron actuator.

The simplified model corresponds to 4 rigid beams with a torsional spring at each anchored end, which form the orthogonal geometry with the center in O. The following equations were used:

(13) $$\omega =\frac{2\pi }{T}\;\mathrm{and}\;\omega =\frac{v}{r}\;\to \;v=\omega r$$

(14) $$v_{E}=\omega_{1}l_{EO},\;v_{F}=\omega_{1}l_{FO},\;v_{out}=\omega_{1}l_{GF},\;v_{in}=\omega_{1}l_{AF}$$

where ω and *v* are angular and instantaneous velocities, respectively; *T* is the period, and all *r* and *l* values correspond to the respective radii.

Considering that velocities, or displacements, of orthogonal beams are near to zero, that means $v_{E}=v_{F}\approx 0$, in congruence with the simulation results. 

Then, the amplification factor of the microgripper, considering both parts, can be approximated by:

(15) $$R_{AMP}=\frac{2x_{out}}{x_{in}}=\frac{2v_{out}}{v_{in}}=\frac{2\omega_{1}l_{GF}}{\omega_{1}l_{AF}}=\frac{2l_{GF}}{l_{AF}}$$

## 3. Results of Theoretical and Numerical Models and Discussion

Simulation results were obtained using Ansys Workbench Software by coupling the tools -> Thermal-electric -> Static Structural, that is, by means of the finite element method. Subsequently, the comparison of the results of the analytical and numerical models is shown in Section 3.

Numerical models correspond to:

1. Validation of the 2-beam microactuator as a U-beam-like microactuator.
2. Validation of the 4-beam microactuator as a U-beam-like microactuator.
3. The V-shaped beam microactuator with 2 and 16 beams.

Finally, the results of the microgrippers corresponding to Models 1 and 2 are given, as well as the amplification factor of Microgripper Model 2.

### 3.1. Thermal Beams Elongation

The lengths of the beams that make up the 2-beam microactuator were parameterized to select the geometric dimensions that allowed the displacement and force parameters generated by the deflection of beams when thermal energy was applied to be improved.

**Figure 5:**
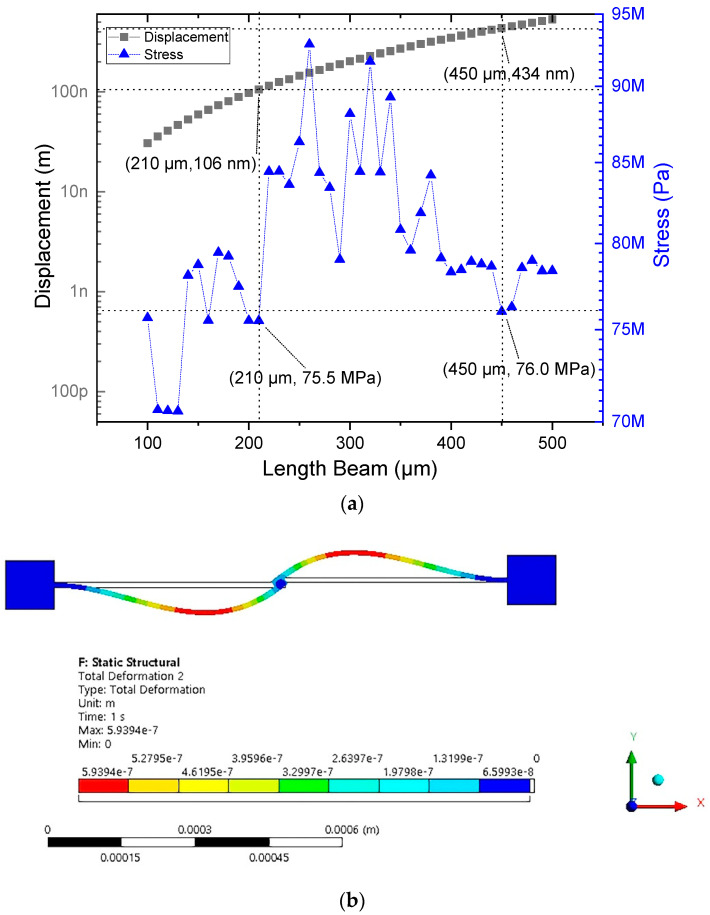
Improved geometric dimensions of the microactuator with 2 beams. (**a**) Parameterization of the beams lengths, and (**b**) simulation of the microactuator with the improved geometrical length.

Figure 5a shows the results of the parameterization; it was observed that a length of 450 µm was an adequate value of displacement. With this length, without considering the dimensions of the junction point, a low stress value was given. Figure 5b shows the temperature distribution, where in the red color can be seen the place where the highest deflection occurred, which was used for the implementation of structural elements (arms of the microgrippers of Models 1 and 2).

**Figure 6:**
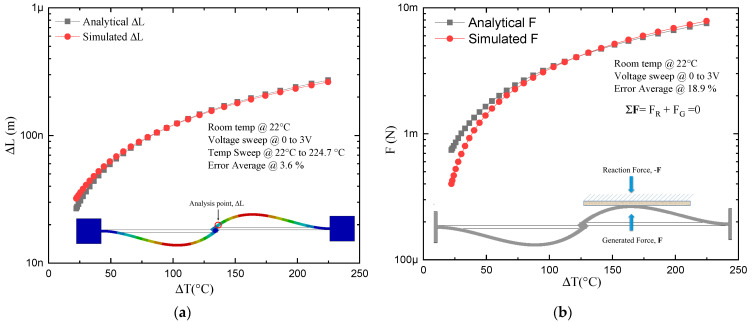
Thermoelectric results of microactuator with two beams. (**a**) Displacement at the middle point. (**b**) Reaction force.

Therefore, the microactuator was considered with two beams embedded at its ends, with the dimensions described in Table 2. For comparison, the elements described in Section 2.2 of the microactuator and Equations (2) and (3) were used, and the displacement results were taken at the middle part of the microactuator or point of analysis (Figure 6a). The acting force was considered directly at the section of maximum deflection of the beam, as can be seen in Figure 6b.

The following conditions were used as part of the operating and simulation conditions of the device: voltage source of 2 V, ambient temperature of 22 °C. Only the parameterization was performed considering a sweep from 0 V up to 3 V to observe in a wide range of values the microactuator performance of the two beams. The same voltage sweep was used for the case of the actuator with four beams.

Table 4 shows a comparison of the results of the theoretical and numerical models, considering a 2 V voltage source. This voltage value was the one considered in the final designs of the microgrippers of Models 1 and 2.

The error margins were very low, so the approximations used were considered adequate, as well as the boundary conditions used in both models.

### 3.2. Electromechanical Modeling of Two Beams vs. U-Beam-like Microactuator

**Figure 7:**
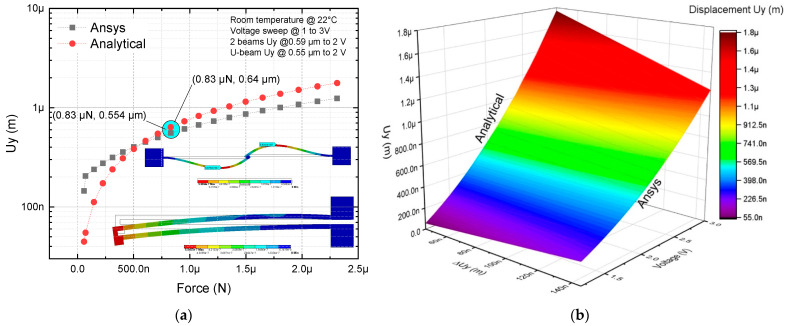
Analytical and numerical models result for microactuators of two beams and U-beam-like actuator: (**a**) 2D and (**b**) 3D representations.

The geometrical relationship of the beams of the two-arm actuator and that obtained by bending both beams, generating an actuator like the U-shaped microactuator, can be seen in Figure 3. For simulation of the U-beam-like actuator, it was fed at one of the fixed ends with a positive voltage and at the other end of the device with a negative potential, like the simulations reported on the U-beam microactuator. One then had one hot arm and one cold arm, so that Equations (4)–(8) could be used. The simulation results are shown in Figure 7, where a voltage sweep was applied from 0 V to 3 V at an ambient temperature of 22 °C.

As can be seen from Figure 7, the analytical and numerical approximations were close, with a greater deviation at the extremes of the curves representing displacement. The maximum values for displacement, force, and stiffness are presented in Table 5. In the case of the two-arm U-beam-like microactuator, for which the analytical approximation was available, it was observed that the maximum error corresponded to the force, with a value of 26.5%, while the minimum corresponded to the displacement in the *Y*-axis, with a value of 12.89%.

### 3.3. Electromechanical Modeling of Four-Beam Microactuator

As can be seen from Figure 6, the analytical and numerical approximations were close, with a greater deviation at the extremes of the curves representing displacement. The maximum values for displacement, force, and stiffness are presented in Table 5. In the case of the two-arm U-beam-like microactuator, for which the analytical approximation was available, it was observed that the maximum error corresponded to the force, with a value of 26.5%, while the minimum corresponded to the displacement in the *Y*-axis, with a value of 12.89%.

**Figure 8:**
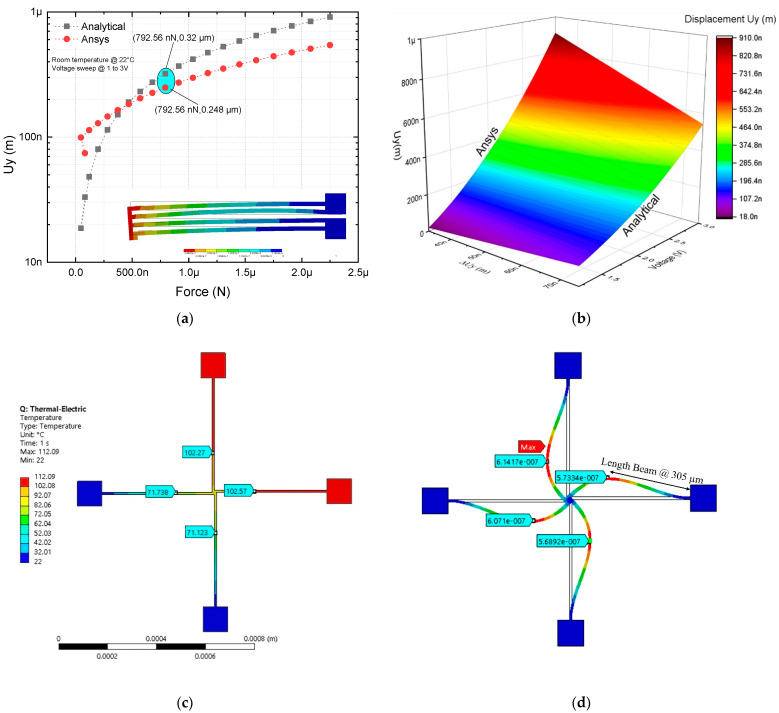
Comparison of analytical and numerical modeling of microactuator with four beams: (**a**) 2D, values of force and displacement at 2V are marked with the blue oval marker, and (**b**) 3D under a voltage sweep from 0 V up to 3 V. (**c**,**d**) Results of temperature and displacement of the rotary microactuator at 2 V, respectively.

For the modeling of the four-arm U-beam microactuator, the distribution of these elements was performed with an analogous arrangement to a U-beam microactuator (Figure 8a,b). In addition, the simulation of the rotary actuator (Figure 8c) was carried out to compare results and validate the four-arm U-beam-like actuator approach.

The parameter values obtained, simulated at 2 V, and from the analytical model, are shown in Table 5.

Considering Equation (9) to calculate the stiffness and the effective length of the rotating microactuator rod, 450 µm + 10 µm of the central part, the following results were obtained from Table 5.

In Table 5, it can be observed that the largest errors correspond to the U-beam-like microactuator with two beams for displacement and stiffness coefficients, at 1.5 V, with values of 31.8% and 68.5%, respectively. For 1.8 V up to 2 V, these errors were considerably reduced. A similar performance was observed for the U-beam-like microactuator with four beams, but with a lower error range, where the largest errors corresponded to the same variables; at the same voltage level, the corresponding values were 21.91% and 42.21%. For the last actuator, force and displacement parameters were within an acceptable range, as they were the within the range of values found in the state of the art reported in relation to the modeling and simulation results because of boundary conditions, temperature effects, and material properties [6,31].

### 3.4. Validation Electromechanical Modeling of V Beam Microactuator

In this section, the focus is on the V-shaped microactuator device, which has been widely studied. Its characteristic equations of operation, which were presented in Section 2, are Equations (9)–(11), which characterize the displacement and force of the microactuator due to electrothermal effects.

**Figure 9:**
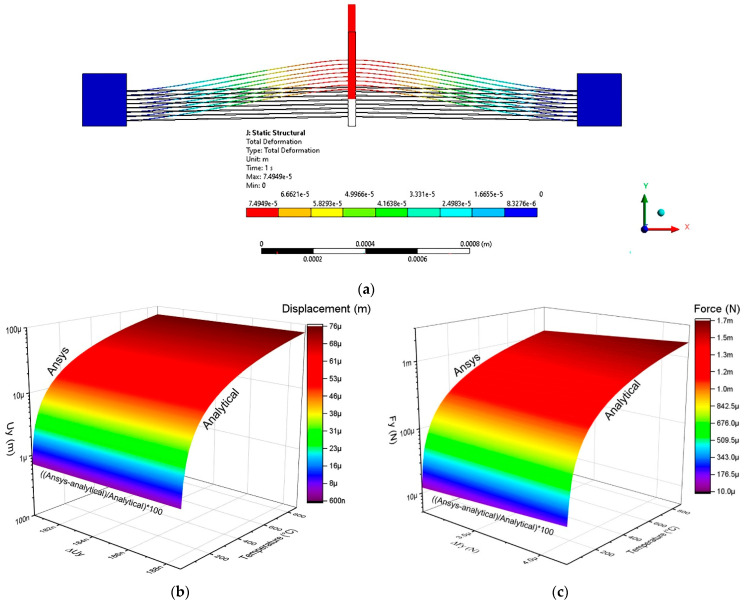
Simulation results of V-shaped microactuator. (**a**) Displacement at 2 V. (**b**) Displacement and (**c**) force responses under voltage sweep from 0 V up to 2 V.

Figure 9a shows the chevron microactuator. When it is fed by a potential difference in its anchors, a heat distribution is generated, which produces the thermal expansion of its beams, producing in turn the linear displacement of the shaft in the *Y* axis. As visualized in the color palette, red corresponds to the largest value. Figure 9b,c show that the analytical and numerical approximations were quite close, since, as mentioned, this is an actuator that has been extensively studied, and a fairly accurate model is already available.

Table 6 summarizes the results of the analytical and numerical models of the displacement, force, and stiffness of the V-shaped microactuator when fed with 2 V; an average temperature of 588 °C and room temperature at 22 °C were considered.

The following boundary conditions were considered: ambient temperature and voltage 0 across the left pad contour. On the other hand, a positive voltage was applied to the right pad contour, as well as an average ambient temperature.

### 3.5. Results by Element Finite Method of Microgrippers Models 1 and 2 

**Figure 10:**
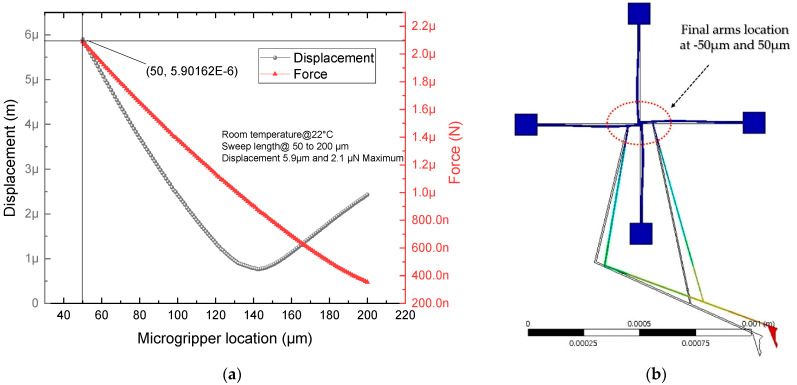
(**a**) Force and displacement parameterization, and (**b**) left section of microgripper Model 1, with the arm locations on the rotary microactuator.

The location of the microgripper arms was determined by parameterizing the position along the horizontal beams (with length of 450 µm) of the rotary microactuator, as can be seen in Figure 10a. The best location points of the arms corresponded to the maximum buckling values of the rotary microactuator beams. The parameterized microgripper design (left section) is shown in Figure 10b. 

**Figure 11:**
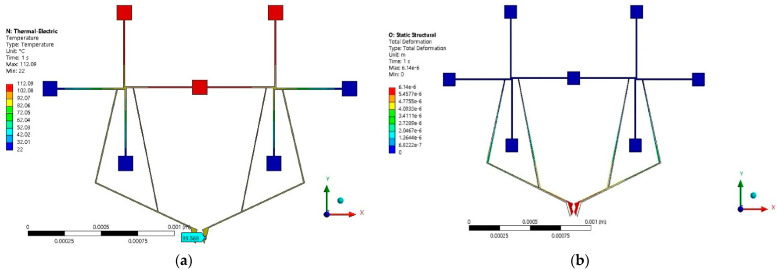
Microgripper Model 1. (**a**) Temperature distribution, and (**b**) displacement at 2 V.

Applying the mirror technique at one of the anchors of geometry shown in Figure 10b generated the complete Model 1, as can be seen in Figure 11. In the red anchors, 2 V was applied. The blue anchors are electrical grounds. All anchors were fixed. The potential generated the closing and opening movement of the jaws, as seen in Figure 11b. 

The simulation results are summarized in Table 7. As can be seen, the reaction force was in the order of µN, so it could be useful in cases where the gripping objects are fragile. The total force was obtained from the constraint of the two gripper jaws, and then the results given in Table 7 corresponded to the force generated by the two gripper jaws.

U total corresponded to the displacement of only one of the jaws, and the inter-jaw displacement was obtained by multiplying this value by two. The results in the three directions are provided to observe if there were some inappropriate displacements that could generate instability in the manipulation. The displacement in the *Z*-axis was residual, which is appropriate.

**Figure 12:**
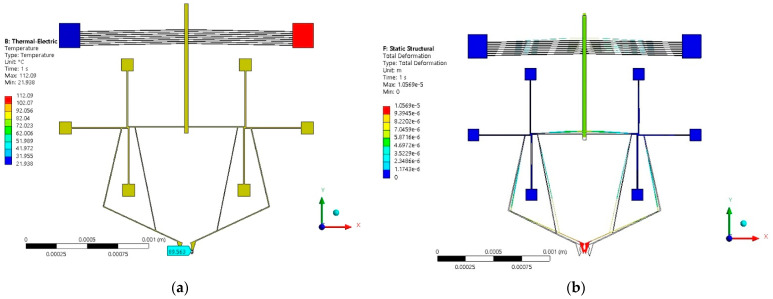
Microgripper Model 2: (**a**) temperature distribution and (**b**) displacement at 2 V.

To improve the response of Model 1 (Figure 11), and with the intention of amplifying or improving the displacement and force parameters, it was decided to use the electrothermal V-shaped beam microactuator as an amplifying element. The chevron was attached to the anchor joining the two rotating microactuators of Model 1, now considering it as a sliding element. Model 2 obtained in this way is shown in Figure 12.

The results of the simulations for Model 2 are given in Figure 12, corresponding to temperature distribution and total deformation. The jaw’s temperature maintained the same value as in the case of Model 1, namely, 89.563 °C, which can allow for a wide range of clamping objects. The values obtained in the simulation of clamping force distribution and displacement are summarized in Table 8.

The simulation was performed with Ansys Workbench software. The Thermal Electric tool was used to apply the potential difference and the ambient temperature condition, with the coupling of a Static Structural to visualize the effect generated by the potential and the displacements. Another Static Structural was coupled to Thermal Electric to obtain the actuation force due to the thermal effect. 

Table 7 and Table 8 show that the total inter-jaw displacement values obtained for Models 1 and 2 were 12.28 μm and 21.2 μm, respectively, and the reaction forces were 8.96 μN and 34.2 μN, respectively. The performance parameters of both microgrippers make feasible their use for different nanoapplications; Model 2 can be used when higher force and displacement are required. 

The simplicity of the structure of Model 1 is remarkable, as it consists of only 14 beams, making it light, simple, and low profile. Model 2, on the other hand, brings together the same elements of Model 1, plus those involved in the chevron actuator, making it more efficient, but with a larger area.

The calculated value of the amplification factor for microgripper Model 2 was obtained by length substitution from Equation (15) and was equal to 4.196. The evaluation of this expression using the input and output displacement values obtained from simulation provided an amplification factor of 3.5. The relative error was equal to 16.58%, which is considered as acceptable.

Technical details about FEA for both microgripper models are given in Table 9.

### 3.6. Comparison with Other Microgrippers

In this subsection, in Table 10, some results from other state-of-the-art of microgrippers are shown to compare the parameters of microgrippers Models 1 and 2 and to observe their competitivity with them.

It was observed that microgripper Models 1 and 2, fed at low voltage levels, compared to the performance results of other microgrippers shown in Table 10, had adequate responses in displacement, while in force they had low levels, which allowed their functionality to be directed to fragile or more sensitive microparticles in their manipulation. The microgripper proposed in [29] was made with polysilicon, and thus the fabrication cost was also different.

Table 11 shows some microparticles or samples with dimensions in the aperture range of the microgrippers Model 1 and Model 2 proposed in this work. 

In addition, in our work, the feasibility of use of the arrangement of rotary actuators was validated. For the micromanipulation of samples and microparticles, it is desirable to have highly accurate and stable micromanipulation systems, which involve microgrippers, high-resolution micropositioners [35], and their respective control systems. It should be noted that micropositioners can even be multi-axis.

## 4. Feasibility of Fabrication

**Figure 13:**
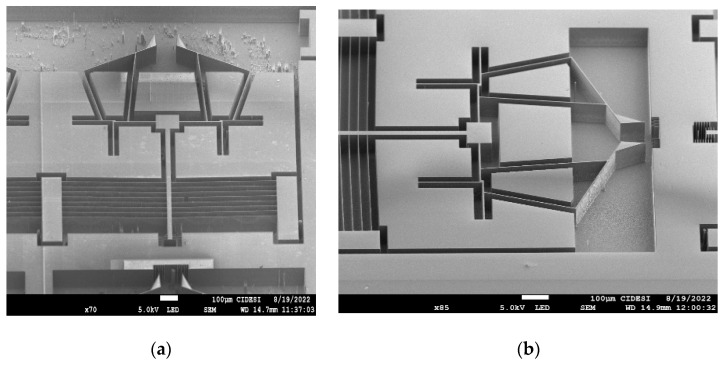
Preliminary results of fabrication in silicon wafers of microgripper Model 2. (**a**) Full geometry, and (**b**) magnification of the microgripper arms and the arrangement of the rotary actuators.

Due to the costs of the silicon on insulator (SOI) process, prior to fabrication on this type of wafer and technology, tests were performed on the feasibility of fabrication using only silicon wafers. It should be noted that fabrication at depths of 70 µm, in laboratories working with shallower depths, is a challenge, so the recipes had to be adjusted for deep reactive ion etching (DRIE) until the required wall verticality was successfully achieved. This team of work was supported by the Centro de Ingeniería y Desarrollo Industrial, CIDESI, Querétaro, for the preliminary manufacturing processes. Fabrication with SOI, once the depth of the structures was validated, is in progress. Figure 13 shows the preliminary results of the silicon wafer fabrication tests, where the well-defined beams and walls can be observed.

## 5. Conclusions

In this article, two models of microgrippers were developed, the arms of which were optimized by parameterization. In microgripper Model 1, an arrangement of two-rotary microactuators, joined by a central anchor, was developed. The theoretical analysis of the rotary actuator was supported by an equivalent U-shaped-like microactuator. The small error values validate the approximation used, especially for the case of two beams. When the number of beams increases, the error also increases.

The position of the microgripper arms of Model 1 was optimized by parameterization, which allowed the position of the point of maximum displacement in the horizontal beams to be determined to achieve the maximum displacement of the microgripper arms.

Subsequently, Model 1 was modified, amplifying its performance with the addition of a V-shaped beam microactuator (generating the microgripper Model 2). In this case, all anchors of the arrangement of rotary microactuators function as physical anchors. A theoretical approach and numerical characterization of the modified microgripper were also performed and validated. 

The total inter-jaw displacement values obtained for Models 1 and 2 were 12.28 μm and 21.2 μm, respectively, and the reaction force was 8.96 μN and 34.2 μN, respectively. The performance parameters of both microgrippers could make feasible their use for different nanoapplications. Model 2 can be used when higher force and displacement are required. A comparison of theoretical and numerical results was performed. The results provide acceptable error values. 

The simplicity of the structure of Model 1 is remarkable, as it consists of only 14 beams, making it light, simple, and low profile. Model 2, on the other hand, brings together the same elements of Model 1, plus those involved in the chevron actuator, making it more efficient, but with a larger area. Important applications for the use of these microgrippers include manipulation of microspheres [21], microassembly [23], and microwires [29]. The temperatures in the jaws of the microgrippers allow for the interaction and manipulation of these types of gripping objects. 

## Figures and Tables

**Table 2:** Geometrical parameters of the microgrippers’ designs (Model 1 and 2).

Element Description	Size (µm)	Element Description	Size (µm)
Anchor length, MTB (Lp_1)	100	Length 6 of HMD (Lg_6)	45
Anchor width, MTB (Wp_1)	100	Width 1 of HMD (Wg_1)	7
Beam length 1, MTB (Lb_1)	450	Width 2 of HMD (Wg_2)	5
Beam length 2, MTB (Lb_2)	470	Length 1 of CM (Lh_3)	1020
Beam width 2, MTB (Wb_1)	10	Length 2 of CM (Lh_4)	1611.5
Total Length 1 of MFB (Lh_1)	1120	Initial gap (IG)	54.53
Total Length 2 of MFB (Lh_2)	1120	Total length microgripper (Lh_5)	1873.2
Length 1 of HMD (Lg_1)	650	Total width microgripper (Lh_6)	2070
Length 2 of HMD (Lg_2)	664.21	Anchor length of MVB (Lp_2)	170
Length 3 of HMD (Lg_3)	704.21	Anchor width of MVB (Wp_2)	200
Length 4 of HMD (Lg_4)	50	Beams width MVB (Wb_2)	3
Length 5 of HMD (Lg_5)	853	Distance between beams MVB (Wb_3)	13
Thickness (t)	10	Beam length MVB (Lb_3)	850.13
Length GF	1049		
**Element Description**	**Size (Degrees)**	**Element Description**	**Size (Degrees)**
Angle 1 of HMD (θ1)	101.9°	Angle 3 of HMD (θ3)	126.3°
Angle 2 of HMD (θ2)	103°	Angle 4 of MVB (θ4)	1°

MTB = microactuator of two beams, MFB = microactuator of four beams, HMD = half microgripper with damping, CM = complete microgripper (design 1), and MVB = V-shaped beam or chevron microactuator.

**Table 3:** Mechanical and electrical properties of materials [7,18,25].

Parameters	Silicon Values
Density, 𝜌 (kg/m^3^)	2329
Thermal expansion coefficient, α (C^−1^)	2.568 × 10^−6^
Young’s modulus, *E* (GPa)	130.1
Poisson’s ratio, ν	0.33
Isotropic thermal conductivity, *κ* (W/m °C)	148
Isotropic resistivity, 𝜌_0_ (Ω × m)	0.00015
Room temperature, *Ta* (°C)	22
Average heat transfer coefficient, *h* (W/m^2^ K)	25
Convection coefficient (W/m^2^ °C)	25

**Table 4:** Parameter results for the microactuator of two beams.

Model Parameters	Temperature at 2 V	Analytical Results	Simulated Results	Error %
Displacement, ∆L (m)	112.09 °C	1.353 × 10^−7^	1.341 × 10^−7^	0.85
Force, F (N)		3.745 × 10^−3^	3.712 × 10^−3^	0.89
Stiffness, $k_{e}$ (N/m)		27680.85	27669.36	0.041

**Table 5:** Parameters results for microactuators.

Device	Voltage (V)	Uy Ansys (µm)	Uy Analytical (µm)	|Error %|	F Ansys (µN)	F Analytical (µN)	|Error %|	Kc Ansys (N/m)	Kc Analytical (N/m)	|Error %|
**U-beam-like microactuator with 2 beams**	1.5	0.315	0.239	31.8	0.312	0.399	21	0.99	1.67	68.5
	1.6	0.358	0.309	15.7	0.404	0.516	21.7	1.13	1.67	47.74
	1.7	0.403	0.384	4.86	0.502	0.641	21.6	1.25	1.67	33.95
	**1.8**	**0.451**	**0.463**	**2.69**	0.606	0.773	21.6	1.34	1.67	24.20
	1.9	0.501	0.547	8.38	0.715	0.913	21.6	1.43	1.67	16.98
	2	0.555	0.636	12.8	**0.830**	**1.06**	**17**	**1.50**	**1.67**	**11.43**
**U-beam-like microactuator with 4 beams**	1.5	0.146	0.114	21.91	0.282	0.380	25.7	1.93	3.34	42.21
	1.6	0.164	0.151	7.92	0.373	0.504	25.9	2.27	3.34	32.03
	**1.7**	**0.183**	**0.190**	**3.6**	0.469	0.634	26	2.56	3.34	23.35
	1.8	0.204	0.231	11.6	0.571	0.771	25.9	2.79	3.34	16.46
	1.9	0.226	0.274	17.5	0.679	0.915	25.79	3	3.34	10.17
	2	0.248	0.320	22.5	**0.793**	**1.06**	**25.18**	**3.19**	**3.34**	**4.49**

**Table 6:** Parameter results for V-shaped microactuator.

Device	Umax (µm) Ansys	Umax (µm) Analytical	Error (%)	Force Ansys (µN)	Force Analytical (µN)	Error (%)	kc Ansys (N/m)	kc Analytical (N/m)	Error (%)
Two beams V-shaped to 2 V with average temperature = 584.5 °C	76.8	70.4	8.35	222.73	203.9	8.47	2.9	2.89	0.34
Sixteen beams V-shaped to 2 V with average temperature = 588.7 °C	74.1	70.9	4.33	1718	1643	4.40	23.18	23.16	0.086

**Table 7:** Parameters values for microgripper Model 1 at 2 V.

Device	Fx (N)	Fy (N)	Fz (N)	Total Force (N)	Ux (m)	Uy (m)	Uz (m)	U total (m)
Microgripper Model 1	3.76 × 10^−10^	8.96 × 10^−6^	7.36 × 10^−11^	8.96 × 10^−6^	5.65 × 10^−6^	2.5 × 10^−6^	5.63 × 10^−9^	6.14 × 10^−6^

**Table 8:** Parameters values for microgripper Model 2 at 2 V.

Device	Fx (N)	Fy (N)	Fz (N)	Total Force (N)	Ux (m)	Uy (m)	Uz (m)	U total (m)
Microgripper Model 2	1.52 × 10^−8^	3.42 × 10^−5^	5 × 10^−10^	3.42 × 10^−5^	9.77 × 10^−6^	6.27 × 10^−6^	6.64 × 10^−9^	1.06 × 10^−5^

**Table 9:** Technical details about FEA in Ansys Workbench for Models 1 and 2 of microgrippers.

Device	Solver Target	Element Type/Mesh/Number of DOF	Face Sizing with Element Size	Inflation			Convergence		Total Mass (kg)
				Transition Ratio	Max Layers	Growth Rate	No. of Total Nodes	No. of Total Elements	
Model 1	Mechanical APDL	SOLID 187/refinement-controlled program	Default	0.272	5	1.2	30712	13006	3.29 × 10^−9^
Model 2							58225	26308	6.25 × 10^−9^

**Table 10:** Comparison with other microgrippers.

Ref.	Device	Material	Feed	Displacement (µm)	Force (µN)	Total Geometrical Sizes
[6]	Microgripper with 12 Z-beams	Poly-Si	80 V	85	6575 at 6 V	3220 µm × 3770 µm × NA
[21]	Microgripper	NA	80 V	≈32	≈100 at 130 V	NA
[29]	Microgripper with two V-shaped actuators	Poly-Si	1 V	19.2	≈0 up to 17,000	≈1200 µm × 960 µm × 10 µm
This work	Model 1	Silicon	2 V	12.28	8.96	1611 µm × 1700 µm × 10 µm
	Model 2	Silicon	2 V	21.2	34.2	2070 × 1873 × 10 µm

**Table 11:** Samples for manipulation.

Microparticles	Sizes	Description	Ref
Clear Polyethylene Microspheres	0.96 g/cc—1 µm to 1700 µm (1.7 mm)	Pure polyethylene polymer microspheres in dry powder form.	[32]
Soda Lime Solid Glass Microspheres	2.5 g/cc—Bulk with Coating Options—3 µm to 75 µm	Silane coating for improved dispersion in aqueous systems and fluorochemical coating.	[33]
Poly (Methyl Methacrylate) PMMA Acrylic Microspheres and Spheres	1.2 g/cc—1 µm to 3.5 mm	Highly spherical uncross-linked clear pure poly (methyl methacrylate) acrylic microspheres and spheres.	[34]

## Data Availability

Not applicable.
